# The origins and evolution of macropinocytosis

**DOI:** 10.1098/rstb.2018.0158

**Published:** 2018-12-17

**Authors:** Jason S. King, Robert R. Kay

**Affiliations:** 1 Department of Biomedical Sciences, University of Sheffield, Western Bank, Sheffield S10 2TN, UK; 2 MRC Laboratory of Molecular Biology, Francis Crick Avenue, Cambridge CB2 0QH, UK

**Keywords:** macropinocytosis, Ras, PI3-kinase, *Dictyostelium*

## Abstract

In macropinocytosis, cells take up micrometre-sized droplets of medium into internal vesicles. These vesicles are acidified and fused to lysosomes, their contents digested and useful compounds extracted. Indigestible contents can be exocytosed. Macropinocytosis has been known for approaching 100 years and is described in both metazoa and amoebae, but not in plants or fungi. Its evolutionary origin goes back to at least the common ancestor of the amoebozoa and opisthokonts, with apparent secondary loss from fungi. The primary function of macropinocytosis in amoebae and some cancer cells is feeding, but the conserved processing pathway for macropinosomes, which involves shrinkage and the retrieval of membrane to the cell surface, has been adapted in immune cells for antigen presentation. Macropinocytic cups are large actin-driven processes, closely related to phagocytic cups and pseudopods and appear to be organized around a conserved signalling patch of PIP3, active Ras and active Rac that directs actin polymerization to its periphery. Patches can form spontaneously and must be sustained by excitable kinetics with strong cooperation from the actin cytoskeleton. Growth-factor signalling shares core components with macropinocytosis, based around phosphatidylinositol 3-kinase (PI3-kinase), and we suggest that it evolved to take control of ancient feeding structures through a coupled growth factor receptor.

This article is part of the Theo Murphy meeting issue ‘Macropinocytosis’.

## 1. Introduction

**Figure 1.**
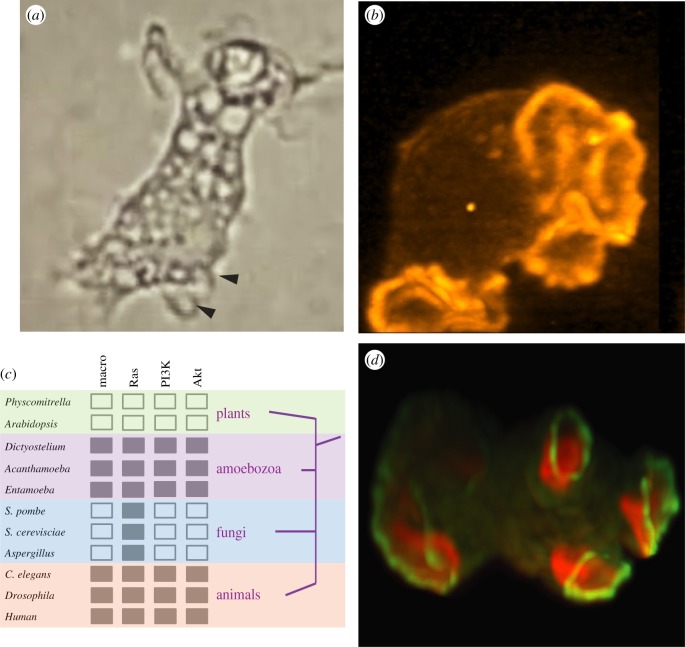
Examples of macropinocytosis and its evolution. (*a*) Macropinocytosis in macrophages. A still taken from a time-lapse movie made by Warren Lewis, who first described macropinocytosis in mammalian cells in 1931 [1]. Vigorous ruffling and macropinosome formation can be seen in the movie—newly formed macropinosomes are indicated by arrows in the figure (added by the authors). The movie was recovered by Dr Joel Swanson, to whom we express our gratitude. (*b*) Macropinocytic cups in a *Dictyostelium* amoeba. The cell is expressing a fluorescent reporter for F-actin and is viewed by lattice light sheet microscopy [3]. The cups are several microns in diameter and are produced at a rate of 1–2 per minute. An axenic strain, Ax2, was used in which neurofibromin (NF1) is deleted and macropinocytosis is much higher than in wild-type cells. Taken from [4]. (*c*) Evolutionary origin of macropinocytosis. Macropinocytic organisms were identified from the literature. The plants and fungi taken as negative are well studied, making it unlikely that macropinocytosis could have been overlooked. Homologous genes were identified by reciprocal BLAST searches and the expected domain structure confirmed using Pfam. The negative organisms have well-annotated genomes, making it unlikely that a homologue would be missed. Note that PI3K orthologues found in *Physocomitrella* and other plants lack Ras-binding domains and thus are not functionally equivalent. The evolutionary relationship among animals, fungi, amoebozoa and plants is shown, with the amoebozoa as a sister clade to the opisthokonts [5]. (*d*) Organization of macropinocytic cups in a *Dictyostelium* amoeba. The macropinocytic patch is revealed by a reporter for PIP3 and the irregular necklace of the SCAR/WAVE reporter around it by HSPC300-GFP. As SCAR/WAVE activates the Arp2/3 complex and is always recruited to the edge of patches, this arrangement should trigger a ring of actin polymerization to form the walls of the macropinocytic cup. Taken from [4].

Macropinocytosis—the non-specific uptake of fluid into large cytoplasmic vesicles—is an actin-driven endocytic process that was clearly described by Warren Lewis in the 1930s [1,2]. His time-lapse movies showed macrophages and tumour cells ruffling and taking in bright droplets of medium at their periphery, which they then transported centripetally (figure 1*a*). The vesicles became progressively stained with neutral red as they acidified and Lewis speculated that the cells were digesting their contents and so feeding. Macropinocytosis was described at about the same time or even earlier in amoebae, such as *Amoeba proteus*, where it could be triggered by dilute salt solutions [6–9]. Much later, laboratory strains of the soil amoeba *Dictyostelium discoideum* were isolated that performed macropinocytosis at a high rate, allowing them to grow in liquid culture [10–12].

Today, tissue culture cells and these *Dictyostelium* amoebae are the main subjects for macropinocytosis research [13,14]. The similarities in how they perform macropinocytosis—particularly the fundamental role of PIP3 (phosphatidylinositol (3,4,5)-trisphosphate in mammals and the functionally equivalent ether-linked inositol phospholipid in *Dictyostelium* [15])—points to a deep evolutionary origin of macropinocytosis in single-celled organisms. Our purpose in this article is to extend this comparison, and explore some of the implications.

## 2. How widespread is macropinocytosis?

In general, macropinocytosis is easily recognizable by light microscopy as the droplets of fluid taken up by cells are readily visible. It has been described in several branches of the amoebozoa. Among the social amoebae, apart from the well-studied *Dictyostelium discoideum* (figure 1*b*) [12,16]*, Polysphondylium pallidum* and *Dictyostelium purpureum* are also proficient, based on their growth in liquid medium [17,18]. Macropinocytosis occurs in other free-living and pathogenic amoebae, including *Acanthamoeba castellanii* [19] and *Entamoeba histolytica* [20,21]. Large freshwater amoebae such as *Chaos carolinense* can perform a morphologically distinct form of macropinocytosis where the fluid is taken into vesicles pinched off from channels penetrating the cytoplasm [9].

Practically any mammalian cell in tissue culture seems capable of macropinocytosis in the right circumstances. Macrophages [1,22] and dendritic cells [23] of the immune system are particularly adept. Others include 3T3, MDCK and HeLa cell lines, where growth factors or activation of the growth factor signalling pathway generally stimulate macropinocytosis [24–27]. Lewis described macropinocytosis in rat and mouse sarcomas [2], and recent attention has focused on Ras-activated tumour cells, such as pancreatic duct adenocarcinoma cells, which can feed by macropinocytosis, thus reverting to the habits of amoebae [28]. Macropinocytosis in tissue culture cells takes different forms, with macropinosomes deriving from either dorsal or peripheral ruffles, which can form cups or flaps that close, or from large circular dorsal ruffles that contract and often leave macropinosomes as their residue.

Macropinocytosis has also been shown in immune cells from both worms and flies. The coelomocytes of *Caenorhabditis elegans* inhabit the pseudocoelom and efficiently clear it of secreted GFP protein [29–31], while endocytosis by *Drosophila* haemocytes is shown by uptake of fluorescent dextrans in primary cultures, where it is independent of dynamin [32], or when the dextran is injected into embryos [33]. The presence of macropinocytic cells in these two organisms opens the possibility of applying powerful genetic methods to the process, as well as studying it *in vivo*.

We are not aware of accepted instances of macropinocytosis in plants or fungi, where in most cases the rigid cell wall would be a hindrance. In particular, it is unreported in the well-studied yeasts *Saccharomyces cerevisiae*, *S. pombe* or in the fungus *Aspergillus nidulans*.

The occurrence of macropinocytosis in multiple species from both the amoebozoa and animals places its evolutionary origin at least as far back as the common ancestor of these two groups (assuming common descent) and implies that it has been secondarily lost from at least some fungal lineages, such as yeasts, that diverged after this common ancestor (figure 1*c*).

Ras-activated PI3-kinases (class-1 PI3-kinase), Ras and probably the protein kinase Akt (which binds PIP3) are central organizers of macropinocytic cups (see below). Genes for these three proteins are present in all macropinocytic organisms examined. Strikingly, in the limited set of non-macropinocytic organisms examined, representing plants and fungi, PI3-kinase and Akt are both absent and Ras is only present in fungi [34]. Thus, it appears that macropinocytosis first evolved in single-celled organisms for feeding, and has been maintained and adapted in animals, but lost from some other branches of the evolutionary tree. Its presence correlates with Ras, class-1 PI3-kinase and Akt.

## 3. Macropinocytosis in mammalian cells and *Dictyostelium* amoebae compared

Given a common evolutionary origin, comparing macropinocytosis in mammalian cells and amoebae should reveal its core, conserved components and mechanisms. Up to the point of sealing an internal vesicle, macropinocytosis is an actin-driven process, sensitive to inhibitors of the actin cytoskeleton and likely to involve a largely generic set of cytoskeletal proteins, such as the Arp2/3 complex and its activators, in both mammals and amoebae. It is the organization of these components in space and time that distinguishes a macropinocytic cup from other structures made by the actin cytoskeleton, such as pseudopods. This organization is a job for small G-proteins and phosphoinositides such as PIP3, as well as the cytoskeleton.

### (a) PIP3 and other phosphoinositides

PIP3 stands out as a key molecule in macropinocytosis in both amoebae and mammalian cells, despite chemical differences in the phospholipid tails between them [15]. Macropinocytosis is strongly inhibited by blocking PI3-kinase activity, either with drugs or genetically [35–37]. In *Dictyostelium*, PI3-kinase mutants take up very little fluid, but are able to make rudimentary cups and similarly in macrophages inhibitors of PI3-kinase do not stop cups from forming, but inhibit their closure.

The most striking feature of macropinocytic cups is the presence at their heart of an intense patch of PIP3. This is very clear in *Dictyostelium* amoebae [38,39] and also seen in mammalian cells [40–42]. PIP3 patches appear to fill the macropinocytic cup up to the lip and have surprisingly sharp edges. In *Dictyostelium*, PIP3 patches can form spontaneously or by splitting and are present throughout the life of the cup, up to the moment it closes. The situation is similar in macrophages, except that the patch only appears when linear ruffles circularize.

PI3-kinase activity is counteracted by the lipid phosphatase PTEN, which converts PIP3 back to PI(4,5)P2, and in whose absence PIP3 levels are elevated. PTEN is excluded from macropinocytic cups in *Dictyostelium*, but recruited to the rest of the plasma membrane [37,43]. Deletion of *PTEN* has opposite effects in mammalian cells and *Dictyostelium*: in mouse embryonic fibroblasts (MEFs) and prostate cancer cells macropinocytosis is enhanced [44,45], whereas in *Dictyostelium* it is almost completely abolished [4]. A key difference is that the *Dictyostelium* experiment used axenic mutants in which PI3-kinase activity is already elevated, giving very high PIP3 levels, which appear to disorganize the actin cytoskeleton. However, both results support the importance of PIP3 for macropinocytosis and perhaps suggest that its level must be carefully regulated.

PI(4,5)P2 levels spike in the macropinocytic cups of macrophages before PIP3 [42]; this has not been described in *Dictyostelium*, though fluid uptake is dependent on PI5-kinase required for PI(4,5)P2 synthesis [46]. After closure of the cup, PIP3 is rapidly lost from the internal vesicle and replaced by PI(3,4)P2 in both *Dictyostelium* and macrophages [30,38,42].

### (b) Ras

Ras also appears to be crucial in macropinocytosis and can activate class-1 PI3-kinases through their Ras-binding domain. In the early days of Ras research, it was found that growth factors both activate Ras and cause cell ruffling and macropinocytosis. Crucially, injection of activated (oncogenic) Ras protein into fibroblasts alone was sufficient to drive ruffling, providing the first direct link between Ras and macropinocytosis [26]. Surprisingly, however, recent work has shown that a triple Ras knock-out cell line can still carry out macropinocytosis, although it depends on PIP3 to do so [44]. At the moment, it is unclear whether in this situation one of its close relatives has substituted for Ras, or whether PIP3 alone can sometimes be sufficient.

*Dictyostelium* has an expanded set of *Ras* genes, making genetic manipulation difficult. However, expressing activated Ras stimulates macropinocytosis in wild-type cells [47], while single and double knock-out mutants show that RasG, RasS and RasB are important for macropinocytosis [4,48–50]. Strong evidence for the importance of Ras comes from increasing its activity by knocking out the RasGAP NF1 (neurofibromin). This results in a 4–10-fold increase in fluid uptake, larger and more frequent macropinosomes and confers the ability to grow in the standard liquid medium [51]. In addition to Ras, its close relative Rap may be involved in macropinocytosis, because knock-down inhibits growth in liquid medium [52] and knock-out of the RapGEF, GflB, reduces macropinocytosis apparently by arresting macropinocytic cups in an extended form [53].

### (c) Macropinocytic patches and downstream effectors

In both macrophages and *Dictyostelium*, PIP3 patches are coincident with patches of active Ras and Rac [4,42], thus giving a signalling region of up to several micrometres in diameter contained within the walls of the macropinocytic cup. This set of signal molecules recruits effector proteins to carry through cup formation and closure. Apart from activating PI3-kinase, Ras may directly regulate cytoskeletal proteins such as the formin, ForC [49]. Rac is also required for macropinocytosis in both mammalian cells and *Dictyostelium* [54–56] and through activation of actin nucleation, gives a link to the cytoskeleton.

PIP3 patches recruit PIP3-binding proteins, some of which are expected to have important roles in macropinocytosis. These include class-1 myosins [57,58], but the classic effector is the protein kinase Akt/PKB. In *Dictyostelium*, Akt is nearly essential for macropinocytosis, providing that a related protein kinase that is not PIP3-dependent but is partially redundant is also eliminated. In these PKB-/PKBR1-cells, PIP3 patches still form but their efficiency of fluid uptake is much reduced [59] (Thomas Williams 2018, personal communication). In mammalian cells the situation is less clear, with inhibition of Akt having little effect on macropinocytosis in macrophages [60], but inhibiting it in stellate cells [61].

### (d) Some differences

In mammalian cells, macropinocytosis is often stimulated by growth factors, though it is constitutive in macrophages and dendritic cells, where it depends on extracellular calcium sensed through a calcium receptor [62]. By contrast, macropinocytosis in *Dictyostelium* does not need receptor stimulation, occurring in isolated cells [56] and in mutants where G-protein coupled receptor (GPCR) signalling is genetically ablated by removal of the Gβ subunit of hetero-trimeric G-proteins. Nor does it depend on extracellular calcium, because it occurs in calcium-free media [56].

Diacyl-glycerol (DAG), produced from PIP3 by phospholipase C (PLC), accumulates in macropinocytic cups in macrophages [42] and inhibiting PLC inhibits macropinocytosis in fibroblasts [63]. However, a similar role for DAG in *Dictyostelium* has not been reported to date, and although PLC is able to feedback and promote PIP3 production, axenic growth is unaffected [64,65].

## 4. The relationship between macropinocytosis, phagocytosis and chemotaxis

As we are learning more about the mechanisms used by cells to generate macropinosomes, it is clear that there is significant overlap with other pathways that rely on the production of membrane protrusions. The similarities between macropinocytic and phagocytic cups are obvious, but local activation of the Arp2/3 complex by SCAR/WAVE also generates the pseudopodia and lamellipodia that drive migration and chemotaxis [66–68]. Both cups and pseudopodia are generated by the same underlying excitability of the cytoskeleton and like macropinosomes, pseudopodia also spontaneously form de novo as well as by splitting [4,69–71]. The differently shaped protrusions thus appear to have evolved by differential regulation of the same core machinery.

**Figure 2.**
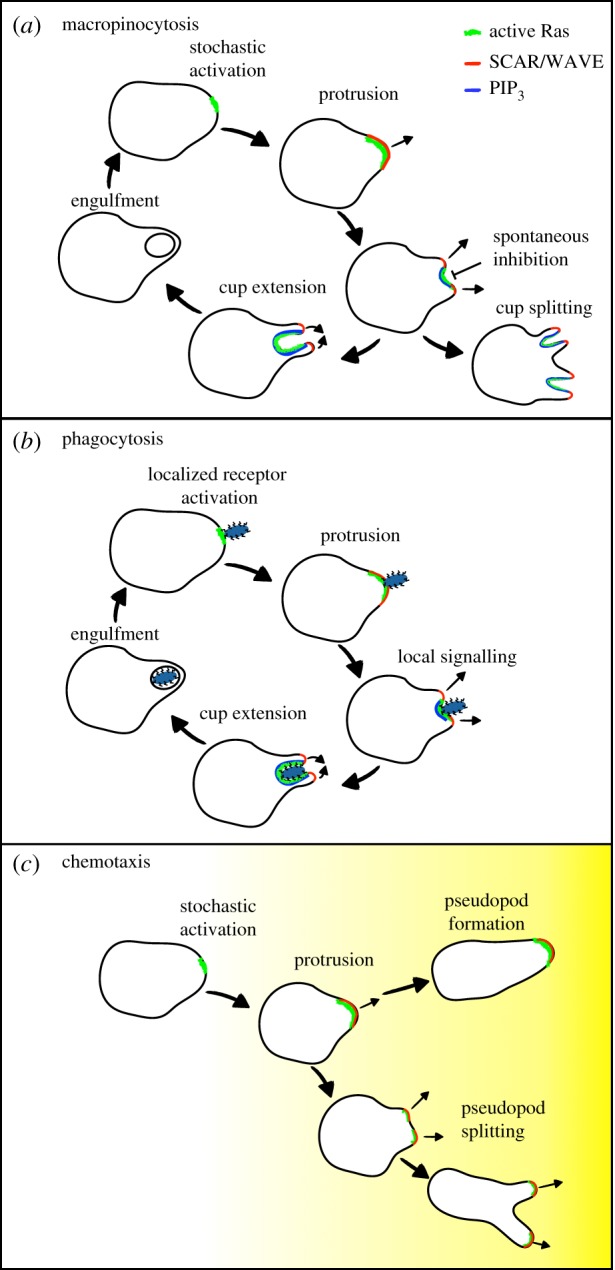
The relationship between eating and migration mechanisms. A shared machinery is used to generate both the cup-shaped protrusions required for macropinocytosis and phagocytosis and pseudopodia that drive migration. This involves small GTPases of the Ras and Rac family, as well as local activation of actin polymerization by the SCAR/WAVE complex. Both (*a*) macropinocytic cups and (*c*) pseudopods form from the spontaneous excitability of the cytoskeleton, and can split. In contrast, phagocytic cups (*b*) are initiated by localized signalling owing to contact with the prey. Whilst each protrusion is driven by SCAR/WAVE activation, cups differ from pseudopods by the presence of a static interior domain, corresponding to the presence of PIP3_._ This self-organizes within a macropinocytic cup, but may be driven by interactions with the target during phagocytosis.

In *Dictyostelium* at least, the formation of the more complex cup shape appears to be an elaboration of the underlying pseudopod machinery. In a simple model, all that is required to convert a pseudopod to a cup is to superimpose a central region where the protrusion is blocked, corresponding to the PIP3 patch (figure 2). In the case of phagocytosis, this is supported by computational models, whereby adhesion to an immovable particle drives a pseudopod to wrap around it [72]. How this would occur in the absence of any particle to form a macropinosome is less clear.

Although the enrichment of PIP3 at the leading edge of chemotaxing *Dictyostelium* cells was initially implicated in chemotaxis [39], there is increasing evidence that PIP3 instead defines the conversion from pseudopodia to macropinocytic and phagocytic cups. While inhibition of PIP3 production almost completely blocks macropinocytosis across evolution, both *Dictyostelium* and neutrophils can still chemotax efficiently when this is done [73,74]. Furthermore, in growing cells with high levels of macropinocytosis, the PIP3-mediated conversion from pseudopodia to cups actually inhibits migration and inhibiting PI3-kinase or physically restricting macropinocytosis enhances chemotaxis of *Dictyostelium* to folic acid [75].

Nonetheless, given a strong enough stimulus, chemotaxis receptors can clearly stimulate localized PIP3 production [39,76,77]. This may be explained by a recent report that both chemotaxis and phagocytosis are mediated by the same receptor that recognizes both diffusible folate and the bacterial surface component lipopolysaccharide [72]. The two processes are thus inextricably linked, with the potential for erroneous signalling if saturated, although whether cyclic-AMP-mediated chemotaxis has similar crossover is not known.

Although phagocytosis and macropinocytosis are highly related and have presumably co-evolved, macropinosomes self-assemble in the absence of localized external signals and receptor activation. If macropinocytic cups are adaptations of pseudopods, the generation of a static cup interior must occur spontaneously. Interestingly, while PIP3 accumulates in macropinocytic cups in all *Dictyostelium* strains, only shallow gradients are seen in pseudopodia of non-axenic strains (Douwe Veltman 2017, unpublished data). It may, therefore, be that formation of a self-sustaining PIP3 patch requires a higher threshold of Ras activation than normal pseudopodia; i.e. low levels of active Ras form pseudopodia while high levels generate cups. The transition to a cup can thus be regulated at the level of GTPase regulatory proteins and stochastic variations in Ras activity.

The dynamic and excitable nature of the cytoskeleton enables it to be flexible and respond to multiple external and intrinsic cues. Although it is impossible to infer evolutionary order, it is easy to imagine how receptor activation could be imposed on macropinocytosis to evolve phagocytosis, or feedback loops allowing phagocytic cups to form spontaneously would allow cells to engulf fluid.

## 5. Processing macropinocytic vesicles

As macropinocytosis probably evolved as a feeding mechanism in single-celled organisms, its initial job was to digest captured proteins or macromolecules to support growth. This role has been maintained throughout evolution to human cancers, but whether other mammalian cells use macropinocytosis for feeding is not known, though clearly worth investigating. Metazoan cells have also adapted macropinocytosis for other purposes, primarily by changing the processing of macropinosomes once internalized. In immune cells, antigen presentation still requires proteins to be fragmented. Therefore, the early stages of macropinosome maturation are broadly conserved between cells and across evolution.

Although many details remain to be resolved, macropinosome maturation shares common elements with other endocytic pathways, such as being regulated by the activities of the Rab family of small GTPases [14,59,78,79]. Classical clathrin-mediated endocytosis (CME) generates endosomes in a completely different way, and largely serves a different purpose—being more a mechanism to turn over specific membrane proteins than feeding. However, both CME and macropinocytosis-derived vesicles accumulate PI(3)P and active Rab5 immediately after internalization to define an ‘early’ compartment, before Rab5 is replaced by active Rab7 to define a ‘late’ stage of maturation and lysosomal fusion [78,80–82]. This indicates a common evolutionary ancestry.

The different endocytic pathways thus have core conserved elements with, for example, Rab5 acting as a generic marker for newly internalized vesicles. While there must also be pathway-specific trafficking steps and a host of other Rabs help to add specificity and variation, there are currently no exclusive markers of macropinosomes, which are frequently identified simply by their size and loading with fluorescent dextran.

### (a) Macropinosome-specific problems

Although there are parallels with other endocytic pathways, macropinocytosis also poses some unique challenges for the cell. First, as the cups lack any clathrin or sorting adaptor protein coats, there is little apparent selectivity in the surface proteins internalized as macropinosomes form [83,84]. Cells undergoing high levels of macropinocytosis will therefore rapidly digest their surface proteins unless they are retrieved before degradation. This is achieved via the combined activities of WASP and SCAR homologue (WASH) and Retromer sorting complex [84]. These complexes are able to sort proteins into recycling vesicles and play multiple roles in endocytic trafficking and are among the first molecules to be recruited to both macropinosomes and phagosomes—having a burst of activity for just the first 2–3 min after internalization.

Macropinosomes are large, aqueous vesicles with a relatively low nutrient content compared to phagosomes. They also have much less membrane in comparison to their contents relative to smaller vesicles: the surface area-to-volume ratio of a 1 µm diameter macropinosome is 10-fold lower than a typical 100 nm endosome. This means that the macropinosomal lumen is relatively hard to acidify by pumps such as the Vacuolar ATPase (V-ATPase) and the concentration of lysosomal hydrolases and their substrates will be low.

**Figure 3.**
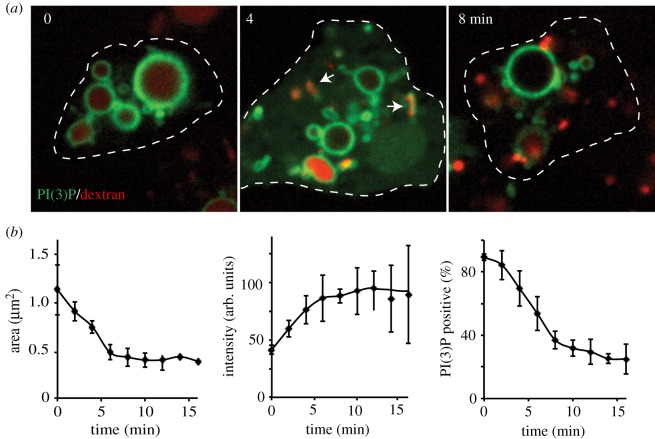
Macropinosome shrinkage and concentration during early maturation. (*a*) *Dictyostelium* amoebae (Ax3) expressing the PI(3)P reporter GFP-2xFYVE [85] were given a 2 min pulse of 0.2 mg ml^−1^ TRITC-dextran (red) before washing and imaging by confocal microscopy. Arrows indicate tubulation of macropinosomes, which occurs while they shrink. The size, fluorescence intensity and degree of colocalization with PI(3)P over time are quantified in (*b*). *N* > 200 vesicles per time point, quantified by automated image analysis (ImageJ). Error bars denote standard deviation. These data are comparable to previous reports in mammalian cells [86,87], indicating that shrinkage and concentration are evolutionarily conserved features of macropinosome maturation.

These problems appear to be solved by the ability of macropinosomes to tubulate and shrink during the first stages of maturation (figure 3). This was reported in the first observations of macropinosomes and has been shown in macrophages and epithelial cells [1,86,87], as well as *Dictyostelium* [88–90]. Importantly, at the same time the vesicle contents become more concentrated, indicating that macropinosomes are shrinking by loss of water and membrane, rather than splitting. This is likely driven by the increased osmotic pressure that must occur upon both tubulation and the fission of small vesicles as they will remove more surface area than volume from the vesicle [91]. Shrinkage and concentration, therefore, appear to be universal parts of macropinosome maturation.

How this shrinkage is achieved is not clear, especially as it occurs at the same time that lysosomes are fusing with the macropinosome and adding membrane. In *Dictyostelium* at least, shrinkage coincides with the presence of PI(3)P on macropinosomes (figure 3*b*), and in mammalian cells was shown to be partly dependent on the activity of the PI5-kinase PIKfyve, which phosphorylates PI(3)P to form PI(3,5)P2 [86]. It seems logical that forming a smaller, more concentrated compartment will aid digestion. However, when shrinkage is reduced upon PIKfyve inhibition, degradation is not significantly affected, and the major defect appeared to be in nutrient extraction, as macropinocytosis could no longer be used to support growth [86]. Although shrinkage is a general feature of macropinosome maturation, the assumption that it aids digestion may not be true in all cases.

### (b) Diversification of the endocytic pathways

**Figure 4.**
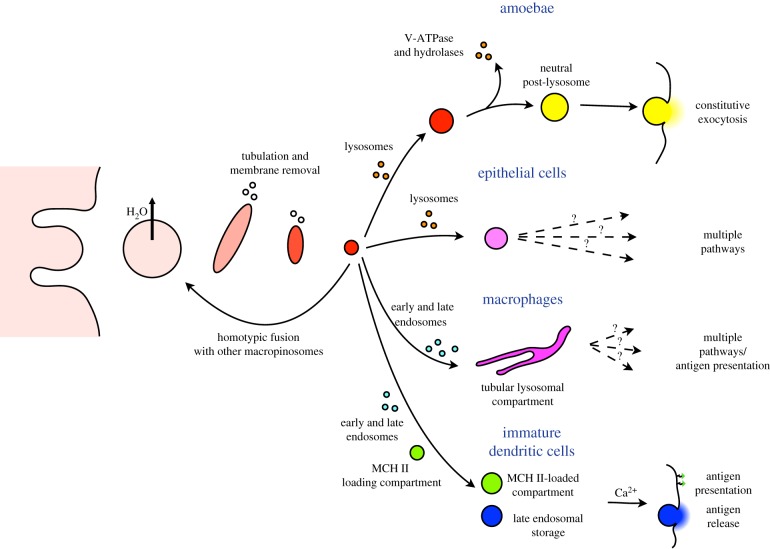
Comparison of macropinosome fate in different cells. The early maturation of macropinosomes and their ability to fuse with each other appears to be universal, but later maturation is more diverse and cell-type specific. In amoebae, macropinosomes appear be independent of other endocytic pathways and undergo a unique post-lysosomal neutralization step prior to constitutive exocytosis. Similar isolation from other pathways has been reported in mammalian epidermal and fibroblast cells, although how the insoluble material is eventually released is not known. In contrast with macrophages and dendritic cells, which use macropinocytosis for antigen presentation, macropinosomes can interact with both early and late endosomes.

The early phases of maturation are highly conserved, but the fate of macropinosomes after they have shrunk has diversified more (figure 4). *Dictyostelium* macropinosomes are able to fuse with one another during the early phases of their transit, but appear to be kept in isolation from other endosomes, as internalized dextrans do not accumulate in any other compartments [88–90].

In higher eukaryotes, the fate of internalized material is more complex and cell-type specific. In particular, there are clear differences in the interactions between macropinosomes and other endocytic pathways. In macrophages, macropinosomes appear to completely assimilate into the lysosomal system after shrinkage and can fuse with clathrin-derived tubular endosomes as well as both early and late macropinosomes [92].

By contrast, in both the human epidermal carcinoma cell line A-431 and NIH3T fibroblasts, though macropinosomes can fuse to each other, they rarely if ever interact with conventional clathrin-mediated endocytic compartments [93,94]. Whether this can be generalized to all epithelial or cancer cells and how this might be achieved mechanistically is unclear, but it indicates fundamental differences in how cells process macropinosomes.

One explanation may be the differing functions of macropinocytosis. Like amoebae, cancer cells use macropinocytosis for feeding. Therefore, the only major prerequisite is to deliver lysosomal components and transport out liberated nutrients. In antigen presenting cells, however, there is an additional requirement to load the digested extracellular proteins onto the Major Histocompatability Complex II (MHC II) molecules before transport to the cell surface. As MHC II is found on a specialized late endosomal compartment, it is essential for macropinosome-derived antigens to interact with the endosomal system at some point. The molecular details of antigen presentation trafficking remain surprisingly poorly understood [95], but it may be that during the evolution of adaptive immunity, immune cells evolved a distinct mechanism to deliver macropinocytic products to the endocytic system absent in other cells.

### (c) Macropinosome efflux

Although digestible components will be transported out and assimilated by the cell, other molecules, such as the dextran-conjugated dyes frequently used to study macropinocytosis, must ultimately be released from cells. In amoebae and presumably other protists feeding by phagocytosis or macropinocytosis, indigestible material is continuously released by constitutive exocytosis [96–99]. This is again best characterized in *Dictyostelium*, where 45 min after internalization, the vesicles transit to a neutral post-lysosomal state. This transition is driven by a second phase of WASH activity, which removes the V-ATPase and hydrolases [100–102]. This is essential for exocytosis and post-lysosomes fuse with the plasma membrane shortly afterwards (figure 4).

There is no evidence for a comparable neutral post-lysosomal compartment in mammalian cells, and therefore macropinosome efflux happens by different mechanisms (summarized in figure 4). Consistent with a complex redistribution across multiple compartments, the release of macropinocytic components from macrophages has complex dynamics, indicating it occurs by at least two pathways with different kinetics [103,104].

In immature dendritic cells, processed antigen from macropinosomes can be both delivered to the MHC II loading compartment, or stored in a late endocytic compartment before being released into the extracellular environment to activate B cells [105]. The details of exocytosis are unclear but it is dependent on both Rab27 and cytoplasmic Ca^2+^ [106]. Remarkably, large increases in cytoplasmic Ca^2+^ are sufficient to stimulate regurgitation of macropinosomes *en masse,* leaving endosomes unaffected, implying this is a specific regulated pathway. Calcium also regulates the fusion of other types of vesicles to the plasma membrane, including lysosomes and synaptic vesicles [107–109]. It therefore seems likely that related mechanisms are employed to deliver the macropinosome-derived vesicles to the surface.

Perhaps surprisingly, little appears to be known about what ultimately happens to indigestible material in non-phagocytic cells. Studies are largely focused on uptake and nutrient liberation, and the prevalence of imaging-based analysis makes studies of efflux dynamics difficult. While early work using C^14^-sucrose as an indigestible marker in fetal lung fibroblasts indicates similarly complex efflux dynamics to that observed in macrophages [104] and fluorescent dextrans are largely lost from A431 cells within 2 h [91], we could find no further mechanistic studies of what ultimately happens to indigestible macropinosome contents. However, the limited information from electron microscopy studies indicates that macropinosomes do not acquire intraluminal vesicles and mature into multivesicular bodies, suggesting an independent fate from classical endosomes [87,110–112]. This may therefore be an interesting subject for future studies.

## 6. Perspectives and questions

Macropinocytosis has been known for approaching 100 years, yet today is much less well understood than the more recently discovered CME: searching for titles containing ‘macropinocytosis’ in the Web of Science yields 367 papers and containing ‘clathrin’ 3229 papers, as of July 2018. This is changing. The realization of the importance of macropinocytosis in the immune system, infection and drug delivery, cancer nutrition and neuro-degeneration [113] has fuelled a complete change in outlook in recent years. As we gain knowledge, several fascinating questions about macropinocytosis come into focus.

### (a) Macropinocytic signalling patches as templates for circular ruffles

A major conceptual problem is to understand how actin can be persuaded to polymerize in a ring under the plasma membrane to form the walls of the macropinocytic cup. This requires organization of actin polymerization over distances of several micrometres, meaning that more than local interactions within the polymerization machinery are required. We suggest that this spatial organization is provided by the patch of PIP3, active Ras and active Rac around which macropinocytic cups form. In *Dictyostelium*, these patches recruit the SCAR/WAVE complex and WASP to their periphery, so in principle activating the Arp2/3 complex and actin polymerization in a hollow ring (figure 1*d*) [4]. How recruitment occurs—whether by movement of the actin effectors to the edge of the patch or unique binding properties there—is not known, nor whether a similar recruitment occurs in mammalian cells.

PIP3 patches must be sustained by unusual dynamics, since PIP3, Ras and Rac are all normally freely diffusible in the membrane. One element is likely to be restricted diffusion at the edges of patches, as described in macrophages [114,115], and another may be positive feedback loops between components of the patches. Active Ras and Rac can still form patches in *Dictyostelium* mutants lacking all class-1 PI3-kinases, suggesting that the autocatalytic kinetics sustaining patches do not require PIP3, although they may be augmented by it [4]. PIP3 patches have been extensively studied under the guise of ‘basal actin waves’ [116] and their formation can be stimulated by the chemoattractant cyclic-AMP in starving cells [117]. High doses of cyclic-AMP induce more patches than low doses, but they are of otherwise the same size and intensity. This suggests patches form by an excitable process, which once triggered proceeds through to completion. The unusually large and intense PIP3 patches of axenic *Dictyostelium* cells, which can be viewed in TIRF microscopy, make an excellent system for discovering the principles of patch formation.

### (b) Closing and sealing macropinocytic cups

To close a macropinocytic cup requires a different form of spatial organization from forming it. As viewed by lattice light sheet microscopy, cups sometimes appear to close by concerted contraction of their lip, suggesting a purse string mechanism [4]. Consistent with a contractile process, myosin-1 proteins are recruited to cups with one class forming a broad ring around the rim [58], and closing phagosomes in macrophages also recruit myosins and are able to exert contractile forces on engulfed particles [118].

The final stage of closing a macropinocytic cup is membrane fusion to seal the cup and form an intracellular vesicle. Little is known of this process at the moment. Because membrane flaps appear able to fuse back to the plasma membrane—though this needs confirming with lattice light sheet microscopy—the mechanism may involve a fusogenic protein such as that recently described in phagosome fusion in *C. elegans* [119], rather than the neck constriction involved in sealing coated pits. Once fusion has occurred it must be signalled so that the macropinosome processing can commence, and again the mechanism is unknown.

### (c) An evolutionary speculation: macropinocytosis and the origins of growth factor signalling

It is a remarkable fact that the core set of proteins organizing macropinocytic cups in *Dictyostelium*—Ras, NF1, Ras-activated PI3-kinase, PTEN and Akt—are the same as those mediating growth factor signalling in metazoa. All are also notable oncogenes or tumour suppressors. The corresponding growth factor receptors are missing in *Dictyostelium* as the genome does not encode receptor tyrosine kinases [120]. We suggest an evolutionary hypothesis to explain this link between growth-factor signalling and macropinocytosis.

We propose that the Ras/PI3-kinase/Akt signalling module evolved in single-celled organisms before the appearance of metazoa and was used to organize their feeding structures, as it is in the amoebozoa to this day. As multicellular cooperation evolved in the branch of phagotrophic organisms leading to metazoa, specialized extracellular digestion arose and most cells were freed of digestive duties but became dependent on others for their food [121]. It was essential to regulate access to this shared resource to prevent selfish appropriation by individual cells, and therefore the activity of the feeding structures in individual cells had to be brought under global control. This we propose was achieved by bringing them under the control of extracellular signals—the growth factors. This would involve linking Ras and PI3-kinase activation to cell surface receptors, so that feeding became conditional on an external signal. The innovation required might be as simple as bringing a critical RasGEF under the control of a receptor. This linkage of feeding structure to surface receptor has already been achieved in *Dictyostelium* for a different purpose, as recent work shows that the folate receptor, which is used to find bacteria, is also capable of triggering their phagocytosis [72,122].

This viewpoint also gives some rationale to the otherwise puzzling linkage of actin dynamics and growth factor signalling. As is well established, growth factor signalling stimulates actin dynamics and macropinosome formation. Amino acids taken up from the medium and transported to endolysosomes by macropinocytosis activate mTORC1 (the mechanistic Target Of Rapamycin complex 1) from there in synergy with a cytoplasmic route via Akt [123,124]. Surprisingly, signalling of less than maximal intensity is sensitive to inhibitors of the actin cytoskeleton, such as the combination of jasplakinolide and blebbistatin [60,125]. It is proposed that this is because macropinocytic cups are triggered by growth factors, and act as signal amplifiers thanks to their intrinsic self-organization and positive feedback loops. As signal amplification depends on their structure, which in turn depends on actin dynamics, signalling becomes sensitive to inhibitors of the actin cytoskeleton [55].

In summary, we see in macropinocytosis an ancient process that evolved for feeding in phagotrophic unicells, and whose original purpose has been at least partially retained in metazoa, with adaptation in immune cells to use the engulfed material for antigen presentation. The traces of its original function can still be seen in the wiring of growth factor signalling, which was added later to gain control of phagotrophic feeding structures in metazoa.

## Data accessibility

This article has no additional data.
